# Integration of heterologous bone matrix associated with polymethylmethacrylate in induced tibial bone defects. An experimental study in rabbits

**DOI:** 10.1590/ACB360704

**Published:** 2021-09-03

**Authors:** Silvio Henrique De Freitas, Alois Foltran Müller, Thaís Ribeiro Fadel, Wellington Henrique Bessi, Renata Gebara Sampaio Dória, Kelly Cristiane Ito Yamauchi, Ricardo De Francisco Strefezzi, Atanásio Serafim Vidane, Carlos Alberto Fortulan, Carlos Eduardo Ambrósio

**Affiliations:** 1 MSc, PhD, Associate Professor. Veterinary Medicine Department (ZMV) - Faculty of Animal Sciences and Food Engineering (FZEA) – Universidade de São Paulo (USP) – Pirassununga (SP), Brazil.; 2 Fellow PhD degree. Postgraduate Program in Animal Bioscience (PPBA), Faculty of Animal Sciences and Food Engineering (FZEA) – Universidade de São Paulo (USP) – Pirassununga (SP), Brazil.; 3 Fellow Master degree. Postgraduate Program in Animal Bioscience (PPBA), Faculty of Animal Sciences and Food Engineering (FZEA) – Universidade de São Paulo (USP) – Pirassununga (SP), Brazil.; 4 MSc, PhD. Department of Veterinary Clinic and Surgery - Faculty of Veterinary Medicine – Cuiaba (MT), Brazil.; 5 MSc, PhD. Department of Clinics - Faculty of Veterinary Medicine - Eduardo Mondlane University - Maputo, Mozambique.; 6 MSc, PhD, Associate Professor. Department of Mechanical Engineering – Universidade de São Paulo (USP), Sao Carlos (SP), Brazil.

**Keywords:** Bone-Implant Interface, Polymethylmethacrylate, Bone Matrix, Bone Cements, Osseointegration, Tibia, Rabbits

## Abstract

**Purpose:**

To analyze and compare the reactions at the interface between the composite, composed of fragmented heterologous mineralized bone matrix (MOMHF) and polymethylmethacrylate (PMMA), and the rabbit’s tibias, through macroscopic evaluation and scanning electron microscopy (SEM) in different periods.

**Methods:**

In this study, 12 New Zealand adult rabbits were used (E1: n = 3, E2: n = 3, E3: n = 3 and E4: n = 3). They had the right tibial defects filled with composite and were evaluated immediately after surgery and at 30, 60, 90, and 120 days.

**Results:**

The composites were incorporated and integrated into the recipient beds in 100% of the cases, defined by the MOMHF osseointegration and the PMMA fibrointegration, with no sign of infection, migration, or rejection.

**Conclusions:**

The behavior of the composites in the recipient beds demonstrates that these biomaterials have the potential to be used in bone defect repairs, offering, thus, better quality of life to the orthopedic patient.

## Introduction

Orthopedic injuries play a prominent role not only in veterinary medicine, but also in human medicine. Therefore, it is not rare for orthopedists to come across bone disorders that require reconstitution. The best clinical option for treating bone defects is to use an autologous bone graft, as it accelerates bone healing. However, there is a drawback of increasing morbidity, pain, surgical and anesthetic time and the risk of injuring normal structures, in addition to limited availability, which may be insufficient for the repair of large bone defects. Because of these drawbacks, other types of bone substitutes, which fill or accelerate the bone tissue formation process, have been researched^1^^,^ 2.

Natural bone implants can be originated from animals of the same or different species. Although biologically inferior to the autograft, they are used for orthopedic repair with good results. The advantage of this alternative is the establishment of a bone tissue bank, with a single donor providing a significant amount of tissue. Bone defects can also be completely and efficiently filled with biomaterials, from natural or synthetic biomimetic sources, such as calcium phosphate cement, hydroxyapatite, polymethylmethacrylate (PMMA) and more^3^^-^ 6.

The implants, both natural and synthetic, should promote osteoinduction, characterized by bone tissue formation from osteoprogenitor cells, osteoconduction, defined by bone growth by apposition of underlying tissue, furthermore being biocompatible, non-carcinogenic, non-antigenic, and with a low inflammatory effect^7^^-^ 12.

The clinical option to repair the bone defects with natural and synthetic biomaterials is highlighted because of their osteoinductive and osteoconductive properties. Moreover, they can provide mechanical support, are easy to acquire, have low cost, do not require any specific tool for the preservation process, and can be produced to fill different sizes of bone defects. However, their manufacture and/or modeling are hard and, for extensive repairs, they need specialized resources and infrastructure^1^^,^ 2 ^,^ 6.

On the other hand, PMMA is almost a bioinert biomaterial, has low cost, easy moldability, high availability, excellent mechanical and physical properties with functional characteristics (gradient, porosity, permeability, impregnation) for *in-situ* use during the surgical act^5^^,^ 13 ^,^ 16 ^,^ 26.

Studies using fragmented heterologous mineralized bone matrix (MOMHF) associated with PMMA to fill bone defects in rabbit tibias have already been carried out and shown quite satisfactory results^2^^,^ 5 ^,^ 9. In addition, during destructive mechanical tests, this composite has shown resistance similar to the one of bone tissue^16^. However, high-resolution morphological analysis of the interface between this biomaterial and the receiving bed is necessary to observe adaptations and properties of inorganic or organic tissues with different densities, which is a fundamental factor to understand the local bone biology^15^^,^ 16 ^,^ 20.

Introduced in 2002, the tibial tuberosity advancement (TTA) technique is used to treat cranial cruciate ligament insufficiency in dogs, with excellent outcomes. After tibial tuberosity osteotomy using an oscillating saw, a predefined size wedge-shaped titanium cage is placed in the bone defect and maintained with plate and screws to provide stability to the tibiofemoral joint^28^. Since the TTA technique development, some modifications were carried out regarding the cage format and composition and also its types of fixation, with good results compared to the original technique^28^^,^ 29. As the tibial tuberosity is under constant action of several forces, the biomaterial intended to fill the bone defect. In addition, it must be biocompatible and have mechanical resistance similar to the bone tissue^16^, so it will not collapse into the defect bone during the incorporation and integration period.

It was proposed in this study to prepare a composite material for bone defect filling, composed of a MOMHF and PMMA, apply it to rabbits tibial defects, and analyze it for osseointegration by observing the reactions into the interface between the composite and the host bone bed.

## Methods

This study was approved by the Ethical Committee on Animal Use of the Universidade de Cuiabá (CEUA/UNIC), number 015/2014, according to National Health Council Resolution no. 196/96.

Twelve New Zealand breed rabbits, white variety, males, 3 months old, weighing between 3 and 4 kg, were divided in four experimental groups: E1 (30 days, n = 3), E2 (60 days, n = 3), E3 (90 days, n = 3) and E4 (120 days, n = 3).

MOMHF was collected aseptically from tibial diaphysis bone fragments of sound adult dogs that came to death due to traumas without signs of infectious-contagious diseases. After the periosteal soft tissue removal, the diaphyseal segment was collected. Following this, the bone tissue was washed in 0.9% saline solution and placed into a sterile glass vessel containing 98% glycerin for a period of not less than 30 days, at room temperature. For use, the preserved bone tissue was fragmented in particles between 1 and 2 mm (MOMHF), which were hydrated in 0.9% saline solution for 10 minutes and dehydrated at room temperature. Then, following the same proportion, PMMA polymer (powder) and PMMA monomer (liquid) were added until reached the molding phase. After that, they were molded using a template (polyamide) of 6 mm of diameter by 15 mm of length. With a manual saw, 1,5-mm thickness discs were obtained, placed into an aluminum container, packed in a paper-plastic pouch and then steam-sterilized at 121°C for 15 minutes following by 15-minute drying period.^2^^,^ 5 ^,^ 16 ^,^ 26

After the right medial tibia clipping, the rabbits were anesthetized with acepromazine (0.1 mg/kg) and tiletamine-zolazepam (20 mg/kg) intramuscularly, followed by a local 2% lidocaine (without vasoconstrictor) anesthetic block.

After antisepsis, a 3-cm skin incision was made using a scalpel, the subcutaneous tissue was dissected, and the biomaterial implantation site was exposed in the proximal medial tibial metaphysis. A 6-mm diameter trephine drill bit, coupled to a low-speed dental motor, was used to create the bone defect.

The tibial bone defects from animals of E1, E2, E3, and E4 groups were filled with the sterilized composite, the periosteum and subcutaneous tissue were approximated with a 4-0 polyglactin 910 suture, and the skin with a 4-0 nylon suture.

In the postoperative period, each animal received five applications of enrofloxacin (10 mg/kg) and three applications of meloxicam (0.2 mg/kg), subcutaneously, once a day, six applications of tramadol hydrochloride (4 mg/kg), subcutaneously, twice a day, and dressing with rifamycin, for 10 days, changed every 24 hours.

The animals were housed individually in cages, fed with commercial rabbit food, water *ad libitum*, and the operated limb was daily evaluated.

The implanted site was then radiographed (50 mA, 0.04 s, and 40 kV), on mediolateral projection, at the immediate postoperative period and at 30, 60, 90, and 120 days after surgery.

At the end of each evaluation period, the animals were euthanized, using the anesthetic protocol described before, followed by cardiorespiratory arrest with propofol and 10% potassium chloride intravenously via auricular vein.

The implanted site on the right tibia was then collected and, after removal of surrounding soft tissues, fixed in 10% buffered formalin for 48 hours. After that, a 10-mm^2^ segment, including the composite and the bone defect, was dehydrated and embedded to PMMA acrylic resin, using a cylindrical silicone rubber mold (13 mm of diameter by 4 mm of length). After the polymerization process, the disc face containing the composite and the receiver bed (sagittal section) was gradually abraded/planned with metallographic sandpaper (no. 400 to 2,000) coupled to the polisher, under irrigation, until its surface became flat and smooth. Afterward, the surfaces of the samples were polished with a 20-mm polishing fabric, coupled to the polisher, and irrigated with alumina solution (0.3 μ)^6^.

The specimens were fixed with carbon conductive adhesive tape and analyzed by scanning electron microscopy (SEM), under a 15-kV acceleration voltage, low vacuum (model TM3000, Hitachi, Hitachi, Ibaraki, Japan), compositional mode. For energy dispersive spectroscopy (EDS) mapping, the images were captured, in tagged image file (TIF) format, by a software connected to the microscope.

As a control, the PMMA present in the composite was used, which, although biocompatible and biotolerable, does not integrate into the bone tissue of the rabbit tibial recipient bed^5^^,^ 13.

## Results

Immediately after surgery, all patients supported the operated limbs, demonstrating that the surgical technique used to implant the composite did not compromise the physical structure of the rabbit’s tibia.

The wounds healed within a period of 12 days, without any signs of reactions that would have suggested rejection or/and infection.

### Radiographic evaluation

At the radiographic evaluation, the composites were in their recipient beds (E1, E2, E3, and E4), without any signs of proliferation or bone lysis.

### Macroscopic evaluation

After removal of the adjacent soft tissues, it was noticed that all composites remained at their recipient beds (E1, E2, E3, and E4), and in the E30 group the recipient beds were fully covered by a thin layer of cicatricial tissue. In E60 and E90 groups, the recipient beds were involved by a much more resistant layer of tissue than those on E30 group. However, in group E120, the recipient beds had a thick layer similar to fibrous tissue, in addition to the cicatricial tissue layer.

### Evaluation by scanning electron microscopy and energy-dispersive X-ray spectroscopy

**Figure 1:**
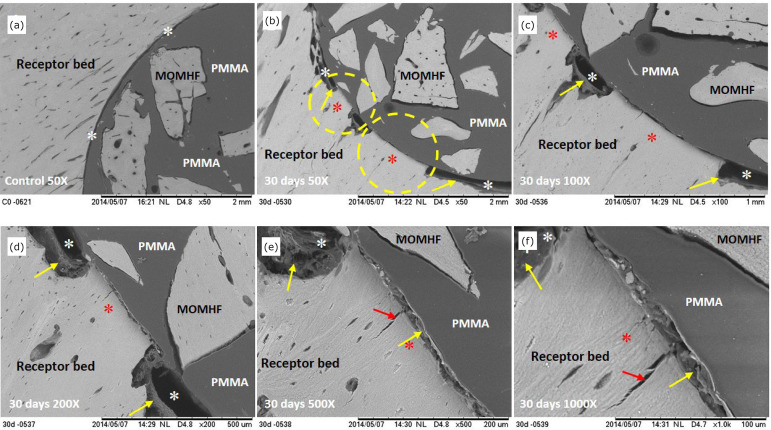
Electromicrographs, obtained by SEM, of the composite in the rabbit’s right tibial recipient bed of the E1 group. **(a-f)** Cortical bone of the recipient bed and MOMHF, interface between composite and recipient bed (white asterisk), neoformed cortical bone (red asterisk and dotted circle), non-mineralized tissue: fibrous tissue with bundles of fibers (yellow arrow), PMMA and bone fissure (red arrow).

**Figure 2:**
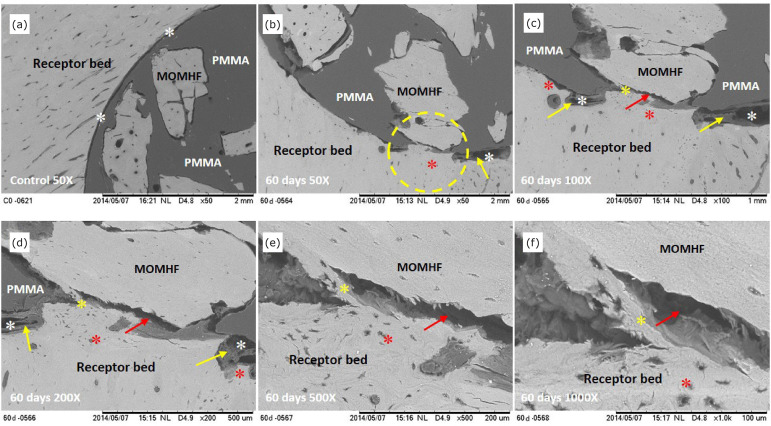
Electromicrographs, obtained by SEM, of the composite in the rabbit’s right tibia recipient bed of the E2 group. **(a-f)** Cortical bone of the recipient bed and MOMHF, interface between composite and recipient bed (white asterisk), neoformed cortical bone (dotted red asterisk), integration of MOMHF with recipient bed (yellow asterisk), non-mineralized tissue: fibrous tissue PMMA and bone fissure (red arrow).

**Figure 3:**
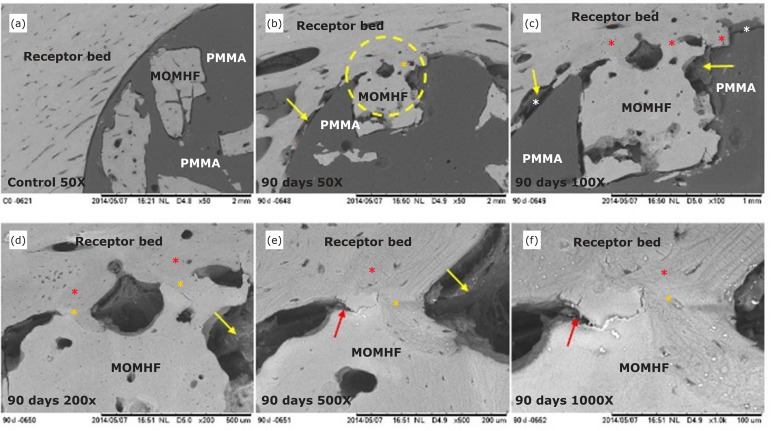
Electromicrographs, obtained by SEM, of the composite in the rabbit’s right tibial recipient bed of the E3 group. **(a-f)** Cortical bone of the recipient bed and MOMHF, interface between composite and recipient bed (white asterisk), neoformed cortical bone (red asterisk and dotted circle), integration of MOMHF with recipient bed (yellow asterisk), non-mineralized tissue: tissue fibrous fibers (yellow arrow), PMMA and bone fissure (red arrow).

**Figure 4:**
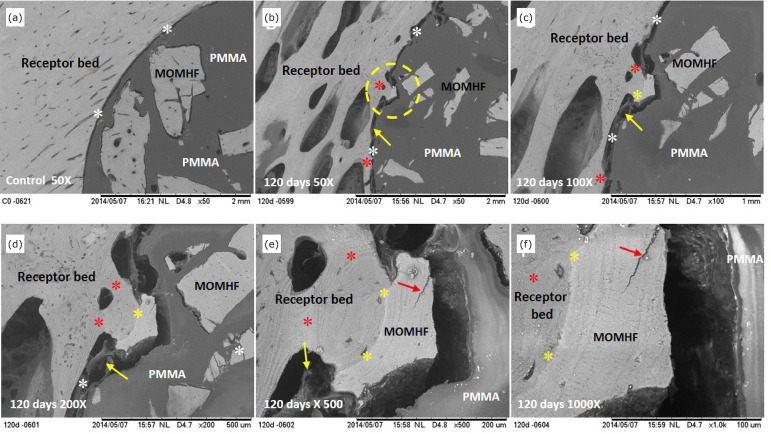
Electromicrographs, obtained by SEM, of the composite in the rabbit’s right tibial recipient bed of the E4 group. **(a-f)** Cortical bone of the recipient bed and MOMHF, interface between composite and recipient bed (white asterisk), neoformed cortical bone (red asterisk and dotted circle), integration of MOMHF with recipient bed (yellow asterisk), non-mineralized tissue: fibrous tissue with fibers bundles (yellow arrow), PMMA and bone fissure (red arrow).

Using the compositional analysis by SEM, it was observed that the implanted composites in the rabbit’s tibia of groups E1, E2, E3, and E4 remained in place at their recipient bed (Table 1). It was also observed that the bone tissue of the recipient bed, the MOMHF, and the PMMA were preserved. Cracks occurred at the osseointegration area, as well as an increasing gap in all sample interfaces (Figs. 1 to 4). In addition, the energy-dispersive X-ray spectroscopy (EDX) mapping analysis of calcium (Ca) and phosphorus (P), both present on the sample surfaces, was performed. Thirty days after surgery (E1 period), the SEM analysis showed, in the interface of the samples, border remodeling of the cortical bone tissue on the recipient bed, with immature bone tissue formation of light gray color (osteoconduction), the presence of non-mineralized tissue, fibrous connective tissue (with fibers bundles parallel to the surface of the dark gray implant in the areas corresponding to the recipient bed), and different cortical bone structures at the PMMA of the composites (Fig. 1).

**Table 1:** The composite behavior on rabbit’s tibial recipient bed.

Groups	Subgroup	Incorporation	Contact with MOMHF	Integration of MOMHF
E1 (30 days)	n.1	+	No	No
	n.2	+	No	No
	n.3	+	No	No
E2 (60 days)	n.1	+	C	I
	n.2	+	C	I
	n.3	+	No	No
E3 (90 days)	n.1	+	C	I
	n.2	+	C	I
	n.3	+	C	I
E4 (120 days)	n.1	+	No	No
	n.2	+	C	I
	n.3	+	C	I

+: incorporation of the composite to the receptor bed; C: contact of MOMHF to the receptor bed; I: integration of the MOMHF to the receptor bed; MOMHF: fragmented heterologous mineralized bone matrix.

**Figure 5:**
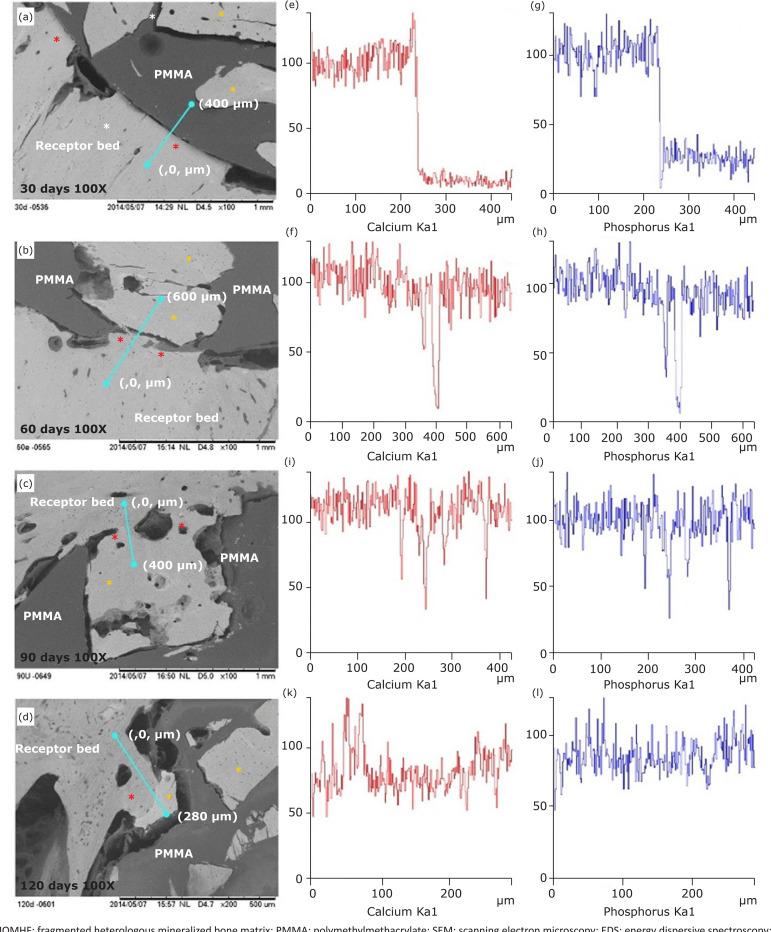
**(a, b, c, d)** Electromicrographs, obtained by SEM, and EDS line scan (blue line) mapping the chemical elements Ca and P of rabbit’s right tibia recipient bed, neoformed bone tissue (red asterisk) and MOMHF (yellow asterisks) after 30, 60, 90 and 120 days. **(e, f, g, h, i, j, k, l)** Note Ca and P concentrations on the region of newly formed bone tissue similar to the recipient bed and to the MOMHF (red asterisk).

By EDX line scan mapping, starting from the recipient bed towards the composite, it was observed that the chemical elements Ca and P remained constant on the surface interface. From this point, there was a decrease concentration of Ca and P, which indicates the beginning of another structure (Fig. 5a-c).

Sixty days after surgery (E2 group), SEM analysis showed, at the sample interface, that the edge of the cortical bone of the recipient bed was remodeling with areas of cortical bone tissue (osteoconduction). It also showed integration in the contact regions of the recipient bed with the composite MOMHF (osseointegration), and the presence of non-mineralized tissue with fibrous connective tissue, in the contact areas between the recipient bed and PMMA (Fig. 2). By EDS line scan mapping, beginning at the recipient bed towards the composite, it was observed that the chemical elements Ca and P remained constant, noticing a decrease over the crack region and then restoration (Fig. 5d-f).

Ninety days after surgery (E3 group), it was noticed, at the interface of all samples, that the edge of the cortical bone tissue at the recipient bed was remodeled, with its presence throughout the bone surface (osteoconduction). We also noted osseointegration between the cortical bone, the recipient bed, and the MOMHF contact area, with the presence of non-mineralized tissue and fibrous connective tissue in the areas between the recipient bed and the PMMA (Fig. 3). Using EDS line scan mapping, starting from the recipient bed towards the composite, it was observed that the Ca and P concentrations remained constant, with some reduce and increase points (Fig. 5 g-i).

A hundred and 20 days after surgery (E4 group), it was observed, at the interface of all samples, that the cortical bone surface of the recipient bed was remodeled, with the presence of cortical bone tissue towards the composite (osteoconduction). It was also observed the contact areas integration of the recipient bed and the MOMHF and the presence of non-mineralized and fibrous connective tissues at the contact areas between the recipient bed and the PMMA (Fig. 4). Using EDS line scan mapping, starting from the recipient bed towards the composite, it was observed that the Ca and P concentrations remained constant (Fig. 5j-l).

## Discussion

The implanted composite will not be completely replaced by bone tissue, since the PMMA, a bioinert material, will be incorporated into the recipient bed, not integrated. Therefore, the analyses at different times were directed to the MOMHF in order to identify integration areas with the receiving bed, which could increase the composite local stability. At the interface, the performance of MOMHF was compared to the PMMA present in the composite, which, even in direct contact with the receiving bed, was not integrated. Thus, as the analyses of this study were restricted to the interface and on a micrometric scale, we concluded that a control group is not necessary, as the PMMA, which does not integrate to the receiving bed, would be sufficient to be compared to the behavior of MOMHF, without compromising the quality of the research and also following the ethical principle of the three Rs (replacement, refinement, and reduction).

The best clinical option for bone defects repairs is autogenous bone tissue. It has cells that stimulate osteogenesis and induce osteoconduction and osseointegration, but it requires two surgical approaches, besides having high cost^5^^,^ 14. For that reason, we have chosen the composite made of MOMHF and PMMA as viable biomaterials, easy to obtain and pack, and demanding only a single surgical procedure for implantation.

The medial proximal metaphysis of the rabbit’s tibia was chosen because of its easy access and thin subcutaneous tissue, reducing, this way, surgical time. In addition, this region has resorptive and osteogenic properties and is the most metabolically active site of this bone^5^^,^ 13.

Although the literature does not recommend a minimum or a maximum time for the permanence of the 98% glycerin preserved composite, it was established in this work a period of not less than 30 days, as suggested by Moreira *et al*.^5^ and Freitas *et al.* 13. Thus, the creation of a bone bank at veterinary surgical centers for use in orthopedic surgeries becomes a feasible procedure^5^.

At the time of rabbit evaluations, no signs of infection or dehiscence at the surgical site were noticed, occurring first intention healing at appropriate period^13^. Also, in the post-operatory period, the tibia resistance was not impaired by the ostectomy^17^.

For making the composites, all the materials used directly to handle MOMHF and PMMA were sterilized. For use, in addition to prior care, the composites were steam-sterilized, being this an essential phase for the surgical procedure^5^^,^ 13 ^,^ 26.

The sterilization process probably did not destroy all the factors, such as bone morphogenetic proteins^27^, that stimulates bone regeneration and osseointegration, present in the MOMHF composite, which had integrated into the recipient bed. However, new studies that seek to identify the main factors related to the MOMHF integration into the rabbit’s tibia recipient bed are necessary.

According to Moreira *et al.* 5 and Kang *et al*.^21^, the permanence of the composite at the recipient bed (Table 1) indicates no rejection, resulting from fibrointegration mediated by PMMA and by the osseointegration of MOMHF, suggesting the composite biocompatibility (Figs. 1 to 5).

Histological techniques are used to analyze the interface between the recipient bed and the biomaterial, and not only the detailed structural characterization of this area, when the material allows very thin slices (<10 μm), as well as the understanding of many cellular and molecular local phenomena^19^. However, for hard tissue sample analysis that does not allow the demineralization process, like the PMMA in our study, the SEM becomes a viable option^20^. For that reason, we chose this technique to evaluate the interface between the tibia recipient bed and the composite (Figs. 1 to 5).

The fibrointegration (i.e., indirect contact by the interposition of the fibrous tissue between the recipient bed and functional implant) has been observed by SEM analysis^15^^,^ 21. With this technique, indirect osseointegration between the recipient bed and the PMMA of all implanted tibia was observed (Figs. 1 to 4). Additionally, we have noticed direct osseointegration between the recipient bed and the MOMHF composite in the tibia of groups E1, E2, E3 and E4 (Figs. 2 to 4, Table 1). This event can also be observed by EDS line scan mapping of Ca and P present in the samples, in which it was observed that ions concentration of these surface elements in the neoformed tissue was very similar to the rabbit bone and to the MOMHF (Fig. 5), demonstrating that it is a mineralized tissue, i.e., bone tissue composed of 30% organic phase and 70% inorganic phase. In this tissue, Ca and P are in Ca phosphate crystals (hydroxyapatite) form and correspond to 95% of the mineralized phase^22^^,^ 23.

At the processing phase for the samples analysis by SEM and EDX line scan mapping, the external surface of both the composite and the recipient bed has been worn by successive sanding. Probably, during this procedure, the MOMHF present on the composite surface may have been removed. However, during the SEM analysis, the absence of contact between the composite and the recipient bed samples E1 (1, 2 and 3), E2 (3) and E4 (1) (Table 1) cannot be considered, once the analysis is punctual and, additionally, the osteoinduction and osteoconduction were present (Figs. 1 to 4).

The fracture lines observed at the contact zones of the recipient bed and the MOMHF (osseointegration site) are due to tissue retraction during the dehydration phase at the sample preparation and by the action of the vacuum at the time of SEM analysis^24^^,^ 25 (Figs. 1 to 4).

MEV analysis was fundamental to evaluate the implanted composites interfaces in the tibial receiving beds. Twelve of the MOMHF and PMMA composites were entirely incorporated into the rabbit’s tibias, and seven were osseointegrated. As this is a punctual analysis, new sites of integration could probably be observed in different areas.

The composite under analysis was satisfactorily incorporated and integrated into the recipient bed of rabbits’ tibia. In addition, it also presented similar mechanical resistance to the bone tissue, as also demonstrated by Catello *et al*.^16^, and may, therefore, be an additional biomaterial option to fill and stabilize bone gaps. Given the potential of this biomaterial, it is intended for future studies to produce wedge-shaped implants (*cage*) to be used as spacers in the modified tibial tuberosity advancement technique (mTTA) for dogs with cranial cruciate ligament disease. We observed in this research incorporation of the composite and integration of MOMHF as a heteroimplant (xenoimplant) in rabbits’ tibia. Probably, this biomaterial will have a better performance, as an alloimplant in dogs’ tibia, to fill and stabilize osteotomies in the mTTA technique. Furthermore, this alternative technique can also reduce the costs of the surgical procedure.

Evaluations of the interfaces between composites of MOMHF and PMMA and recipient beds using microtomography, which allows analysis in different planes, could demonstrate more information regarding the interaction of this biomaterial with the implantation site.

The main observed limitations during the experiment were: the availability of a healthy bone tissue donor, because, unlike human medicine, which has bone tissue banks with strict control, veterinary medicine in Brazil does not have this kind of structure; the appropriate manipulation and production room for natural and biological biomaterials equipped with a safety cabin; the biomaterial implantation site standardization, which was, in this case, the rabbit’s medial proximal tibia; and the availability of appropriate orthopedic and surgical instruments to perform the bone failures.

## Conclusion

The composite constituted by MOMHF and PMMA was incorporated and integrated into the recipient bed in 100% of the cases, as evidenced by the MOMHF osseointegration and PMMA fibrointegration, with no signs of infection nor migration and/or rejection, demonstrating that it is biocompatible and can, therefore, be an additional option for bone defect repairs.

For future studies, modern resources of tridimensional image of the bone lesion and digital impression of the composite will be used, so a greater interaction between the biomaterial and the recipient bed can be possible.
